# Safety of pericardiocentesis in pulmonary arterial hypertension: a systematic review

**DOI:** 10.3389/fcvm.2025.1610419

**Published:** 2025-08-18

**Authors:** Maria Jose Cabada-Garcia, Jahir Rodriguez-Rivera, Carlos Jerjes-Sanchez, Mauricio Castillo-Perez, Paola Gutierrez-Gallegos, Ana Lucia Martinez-Rodriguez, Enrique Paredes-Gutierrez, Renata Claudia Quevedo-Salazar, Humberto De Leon-Gutierrez, Oscar David Lopez-Cortes, Jathniel Panneflek, Raul Monjaras-Alvarado, Jesus Antonio Moron-Mosso, Jaime Guillermo Gonzalez-Medina

**Affiliations:** ^1^ Tecnológico de Monterrey, Escuela de Medicina y Ciencias de la Salud, Monterrey, Nuevo León, México; ^2^ Fellow of the General Directorate of Quality and Health Education, Ministry of Health, Mexico City, México; ^3^ Instituto de Cardiología y Medicina Vascular, TecSalud, Escuela de Medicina y Ciencias de la Salud, Tecnológico de Monterrey, San Pedro Garza García, Nuevo León, México

**Keywords:** pulmonary hypertension, pulmonary arterial hypertension, pericardial tamponade, pericardial effusion, pericardial decompression syndrome, mortality

## Abstract

**Introduction:**

Controversy persists regarding the use of pericardial effusion drainage in patients with pulmonary arterial hypertension (PAH), as several studies report high rates of post-procedure morbidity and mortality.

**Methods:**

We conducted a systematic review to evaluate the safety of pericardiocentesis (PC) in patients with PAH and a large or hemodynamically significant pericardial effusion. We focused on studies involving patients with PAH who presented with a large or hemodynamically significant pericardial effusion and underwent PH. Our primary objective was to evaluate the incidence of major periprocedural complications, and our secondary objectives were to identify the clinical presentation and echocardiographic findings.

**Results:**

We identified 35 patients across 16 studies. Connective tissue disease was the most common etiology of PAH. Drainage strategies during PC differed across studies. The overall mortality rate was 20%, and we identified pericardial decompression syndrome in 14% of patients. Dyspnea and peripheral edema dominated the clinical presentation. Echocardiographic findings of cardiac tamponade, particularly left-sided chamber collapse, appeared more frequently.

**Discussion:**

PC in patients with PAH carries a heightened risk of pericardial decompression syndrome and mortality. However, careful patient selection, echocardiographic guidance, gradual decompression, and continuous hemodynamic monitoring during the procedure may help improve outcomes.

**Systematic Review Registration:**

PROSPERO 585310.

## Introduction

Pulmonary arterial hypertension (PAH) is a vascular disease characterized by a progressive increase in pulmonary vascular resistance and pulmonary arterial pressure, leading to right ventricular (RV) dysfunction, heart failure phenotypes, and premature death (1). Pericardial effusion (PE) occurs in up to 25%–29% of patients with PAH (1–3), with higher rates observed in PAH related to connective tissue disease (CTD) (4, 5). The Mexican REMEHIP registry (1) identified a 10% prevalence of PE in patients with PAH, and the REVEAL (6) and REHAP (7) registries associated PE with an increased risk of death in this subgroup. PE may occur due to the disruption of lymphatic and venous drainage around the heart secondary to the increased right atrial (RA) pressure seen in PAH. However, serositis may also cause PE independently, especially in CTDs. Recently, myocardial edema has been experimentally identified as a stage preceding the development of PE, reflecting hemodynamic deterioration prior to the clinical onset of PE (8). PE is independently associated with worse prognosis (9) and increased mortality (2, 10–13), regardless of the presence of other risk factors. Additionally, it is a poor prognostic factor in the three-strata risk-assessment model in patients with PAH (2). A direct proportional relationship has been observed between the severity of PE and the risk of mortality, underscoring its importance as a poor prognostic factor and a predictor of more intensive treatment.

Although the clinical significance of PE is clear, guidelines for managing this specific population remain poorly defined. An enlarged PE induces significant hemodynamic instability without treatment, particularly when associated with RV dysfunction. Paradoxically, relieving the effusion may result in a sudden increase in venous return and transmural pressure, leading to RV decompensation and circulatory collapse attributed to pericardial decompression syndrome (PDS) (14, 15). Existing evidence regarding the safety and risk-benefit ratio of pericardiocentesis (PC) in patients with PAH remains controversial, complicating clinical decision-making (13, 14, 16–18). The only available systematic review addressing the role of pulmonary hypertension (PH) in the development of cardiac tamponade does not explicitly assess the outcomes of PC (15). Given the lack of conclusive evidence (10, 14), we conducted the first systematic review to evaluate the safety of PC in patients with PAH associated with large or hemodynamically significant PE.

## Methods

### Systematic review protocol and study design

We conducted a systematic review adhering to the PRISMA statement (19) and registered the protocol in PROSPERO under ID number 585310. The primary objective of this study was to evaluate the safety of PC in patients with PAH presenting with a large or hemodynamically significant PE by assessing the incidence of major periprocedural complications. The secondary objective was to identify the clinical and echocardiographic findings associated with large or hemodynamically significant PE in patients with PAH.

### Search strategy and data sources

We conducted an electronic search of PubMed, Scopus, Web of Science, OpenGrey, and Google Scholar for case reports, case series, case-control studies, randomized clinical trials, registries, and prospective and retrospective studies published until July 2024 (Supplementary Appendix S1). The team simplified the final search strategy to refine the scope of studies using keywords and MeSH terms (Supplementary Appendix S2). We employed snowball sampling to minimize lost reports and utilized controlled vocabulary (Supplementary Appendix S3). We collected the articles using the Zotero reference manager software.

### Eligibility criteria

We included studies involving patients with PAH associated with a large or hemodynamically significant PE who underwent PC. The diagnosis of PAH required confirmation through right heart catheterization. We excluded systematic reviews and studies, including groups 2, 3, 4, or 5 PH; those that did not report patients' clinical course; studies with patients under 18 years of age; those primarily focused on trivial or small PE; and cases where PE was due to malignancy, trauma, or recent cardiac surgery.

### Study selection and data extraction

Two investigators performed the search strategy and initial screening using the Rayyan software (22). Our team consisted of cardiologists, residents, and medical students who underwent proper training regarding PAH from a cardiologist with extensive expertise in the field. After eliminating duplicates, investigators independently identified potentially eligible studies by examining the titles and abstracts, and subsequently obtained full articles to assess adherence to eligibility criteria. A third researcher with experience in the field resolved any disagreement regarding study inclusion. The investigators then independently extracted and analyzed data through a double-data extraction process into an online collaborative database with controlled access, which was constructed to consider the variables of interest (Supplementary Appendix S4).

### Risk of bias

We reviewed various databases, including the grey literature. We used hand-searching and snowballing methods (23) to ensure broad study coverage. There were no language restrictions. Two researchers independently performed the screening, eligibility analysis, and data extraction. We held regular discussions to review the extracted data, resolve disagreements through consensus, and systematically enter the data into the database. Based on the CONSORT guidelines and the Newcastle-Ottawa Quality Assessment Form for Cohort Studies, we designed a data quality strategy to evaluate the included studies.

### Data synthesis and analysis

We conducted a formal narrative synthesis of the demographic, clinical, echocardiographic, right heart catheterization, and periprocedural complications information of the included studies. We used summary statistics for continuous and categorical variables according to their types and distributions. We obtained the reported frequency and percentage for continuous variables, as well as the weighted average and standard deviation for continuous variables, in studies with more than one patient.

### Definitions

PAH: mean pulmonary arterial pressure (mPAP) ≥20 mmHg at rest, measured by right heart catheterization, pulmonary artery wedge pressure ≤15 mmHg, and pulmonary vascular resistance >2 Wood units (20).

PE: an abnormal accumulation of pericardial fluid in the pericardial cavity (trivial: seen only in systole, corresponding to <50 ml of pericardial fluid; small: <10 mm, corresponding to 50–100 ml; moderate: 10–20 mm, corresponding to 100–500 ml; large: >20 mm, corresponding to >500 ml; very large: >25 mm, corresponding to >700 ml) (21).

Cardiac tamponade: cardiac compression due to fluid accumulation within the pericardial sac, resulting in impaired diastolic filling of the ventricles associated with clinical instability (hypotension, respiratory distress, etc.) (24).

PDS: acute hemodynamic deterioration and/or pulmonary edema resulting from sudden ventricular dysfunction that occurs following an otherwise uncomplicated PC (25, 26).

Mayor periprocedural complication: death, major bleeding, traumatic injury to cardiac structures, injury to surrounding structures, significant hemodynamic compromise, or circulatory collapse requiring cardiopulmonary resuscitation, intraprocedurally or up to 30 days post-procedure (16).

See the Supplementary Material (Appendix S3) for additional definitions.

## Results

**Figure 1:**
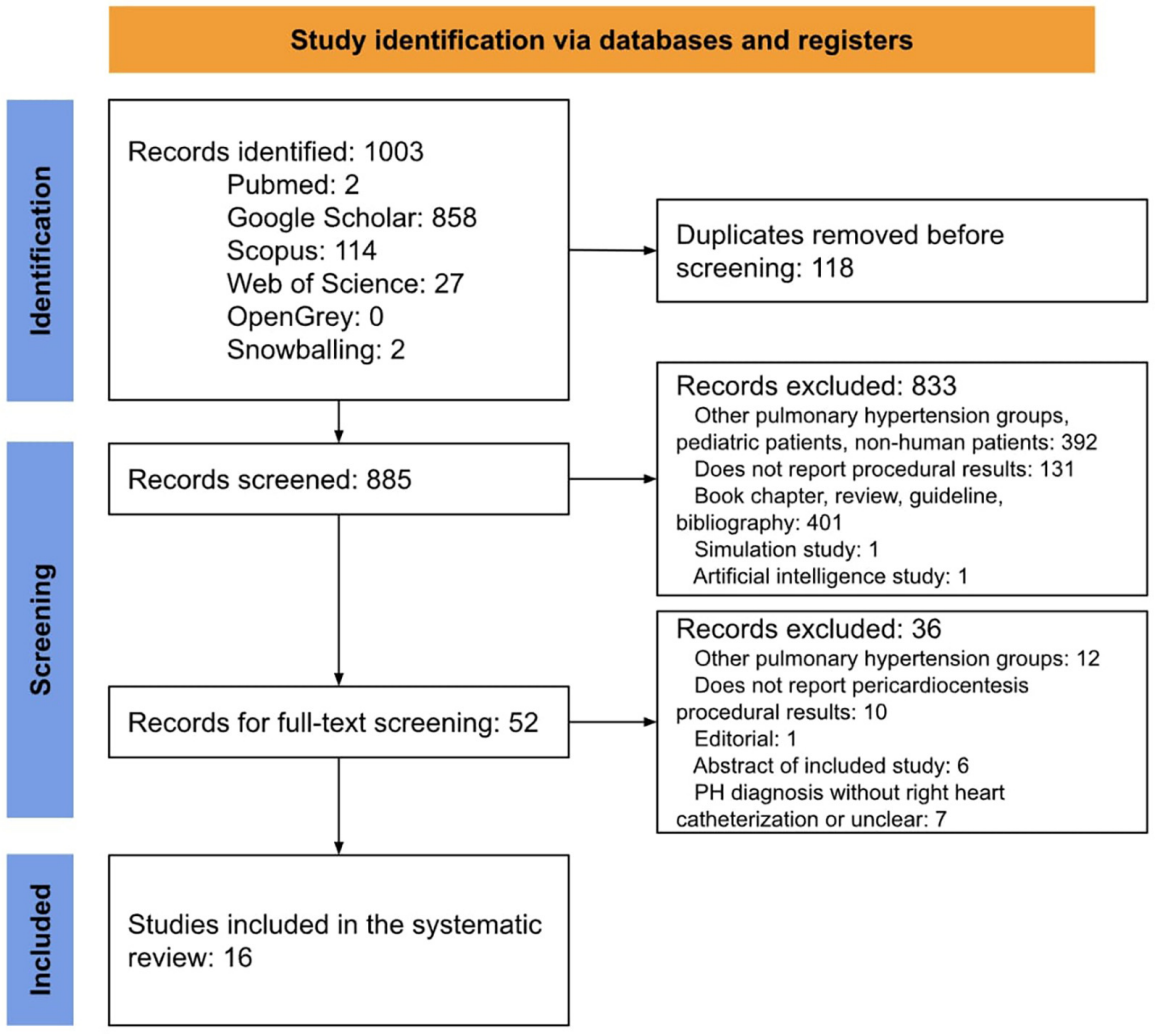
PRISMA flow diagram.

We systematically reviewed PubMed, Scopus, OpenGrey, Google Scholar, and Web of Science on September 12, 2024. Figure 1 shows the PRISMA Flow Diagram. The initial search yielded 1,003 articles, of which 885 remained after duplicates were removed. Two investigators independently screened the titles and abstracts, eliminating 833 articles. The two investigators analyzed 52 articles through full-text screening and included 13 studies that met the inclusion criteria. We contacted the authors of seven additional articles during full-text screening because of the unclear diagnostic methods for establishing PAH. Three authors confirmed the diagnosis of PAH using right heart catheterization (17, 27, 28). We performed double-data extraction of the 16 included articles (13, 14, 16–18, 27–37) (Supplementary Table S1–S7). The included articles are one prospective cohort, three retrospective cohorts, two case series, six case reports, and four abstracts. The risk of bias assessment identified all studies except one (13) as of poor quality, primarily due to the study design (Table 1). 62.5% of the studies included patients <55 years, and most were female. The most common PAH group was associated with CTD, mainly systemic sclerosis (SSc). Most studies included patients receiving PAH therapy. Dyspnea was the predominant clinical presentation reported across most studies, followed by peripheral edema (Table 2).

**Table 1:** Quality assessment.

ID	Year	Author	Study design	*N*	Risk of bias
1	2008	Frantz, R.P	Prospective cohort	1	Poor quality
2	2021	Ansari, Z	Case report	1	Poor quality
3	2024	Chen, E	Abstract	1	Poor quality
4	2022	Laimoud, M	Case report	1	Poor quality
5	2020	Yo, S	Case report	1	Poor quality
6	2011	Fenstad, E.R	Abstract	1	Poor quality
7	2013	Fenstad, E.R	Retrospective cohort	14	Fair quality
8	2024	Singh, A	Case report	1	Poor quality
9	2007	Hemnes, A.R	Case series	4	Poor quality
10	2015	Batal, O	Retrospective cohort	2	Poor quality
11	2019	Case, B	Retrospective cohort	3	Poor quality
12	2021	Weaver, M	Abstract	1	Poor quality
13	2019	Ruopp, N	Case report	1	Poor quality
14	2023	Ruge, M	Case series	1	Poor quality
15	2017	Worsham, C.M	Abstract	1	Poor quality
16	1989	Frey, M.J	Case report	1	Poor quality

**Table 2:** Study design and patient characteristics of studies on PAH patients who underwent pericardiocentesis.

Author	Study design	Patients	Age	Female (%)	PAH etiology	PAH therapy (%)	Clinical presentation
Frantz et al. (29)	Prospective cohort	1	—	—	—	100	Dyspnea
Ansari et al. (30)	Case report	1	32	100	Associated with CTD (SSc)	100	Tachycardia, shock, oxygen desaturation, peripheral edema, altered mental status, dyspnea, faint heart sounds, JVD
Chen et al. (31)	Abstract	1	61	100	Idiopathic	100	Hypotension, peripheral edema, dyspnea
Laimoud et al. (27)	Case report	1	28	100	Idiopathic	100	Tachycardia, shock, vasopressor requirements, oxygen desaturation, peripheral edema, palpitations, dyspnea, JVD
Yo et al. (32)	Case report	1	37	100	Associated with drugs and toxins	0	Normotensive, dyspnea
Fenstad (33)	Abstract	1	54	100	Associated with CTD (limited scleroderma)	0	Peripheral edema, presyncope, dyspnea, loud S2
Fenstad et al. (13)	Retrospective cohort	14	54 ± 9	42.9	71% Associated with CTD	-	Tachycardia, dyspnea
Singh et al. (14)	Case report	1	33	100	Associated with CTD (rheumatoid arthritis and SSc overlap syndrome)	100	Tachycardia, shock, vasopressor requirements, peripheral edema, dyspnea, chest pain, faint heart sounds, JVD
Hemnes et al. (17)	Case series	4	38.75 ± 3.5	75	- 25% Idiopathic - 25% Associated with drugs and toxins - 25% Associated with CTD (scleroderma) - 25% Associated with portal hypertension	100	25% Vasopressor requirement
Batal et al. (34)	Retrospective cohort	2	—	—	Associated with CTD (50% SSc, 50% scleroderma)	50	—
Case et al. (16)	Retrospective cohort	3	60.67 ± 7.5	100	66.67% Associated with CTD (50% systemic lupus erythematosus, 50% rheumatoid arthritis)	—	—
Weaver et al. (35)	Abstract	1	57	100	Associated with CTD (scleroderma type 1)	100	Peripheral edema, dyspnea
Ruopp et al. (18)	Case report	1	60	100	Associated with HIV infection	100	Shock, peripheral edema
Ruge et al. (36)	Case series	1	51	100	Associated with CTD (antisynthetase syndrome and possible Sjogren's syndrome)	0	Tachycardia, oxygen desaturation, peripheral edema, dyspnea, JVD, pulsus paradoxus
Worsham et al. (28)	Abstract	1	31	100	Associated with CTD (SSc and systemic lupus erythematosus overlap syndrome)	100	Presyncope, dyspnea, chest pain
Frey (37)	Case report	1	44	100	Idiopathic	—	Normotensive, peripheral edema, dyspnea, loud S2, JVD, pulsus paradoxus

—, not reported; PAH, pulmonary arterial hypertension; CTD, connective tissue disease; SSc, systemic sclerosis; JVD, jugular venous distention.

Studies qualitatively classified the effusion size. Various studies performed echocardiography-guided PC (13, 14, 16, 18, 27, 33) and PC with real-time invasive hemodynamic monitoring (14, 18, 35). The total amount of pericardial fluid drained across the studies ranged from 80 to 2400 ml. The drainage strategy differed, with most studies performing gradual drainage over several hours or days (Table 3). Nine studies reported pre- and/or post-PC right heart catheterization data (Table 4), describing significant decreases in RA pressure, mean pulmonary artery pressure, pulmonary vascular resistance, intrapericardial pressure, and higher cardiac index values following the procedure. PDS was noted when studies reported PDS, hemodynamic instability, hypotension, shock, circulatory collapse requiring cardiopulmonary resuscitation, or vasopressor requirement following uncomplicated PC. PDS was the most frequent complication in five (14%) patients (17, 27, 30, 31, 34). Two case reports (27, 30), one abstract (31), one retrospective cohort (13), and one case series (17) (Table 5) reported 30-day all-cause mortality after the intervention. The remaining studies reported marked improvement in clinical status or discharge without complications. Six studies (13, 16, 17, 29, 34, 36) included patients who met the inclusion criteria from within a larger cohort. In these cases, we extracted data from the included patients for our review, and the Supplementary Table shows the study results for all patients in that study (Supplementary Table S8).

**Table 3:** Pericardial effusion and pericardiocentesis characteristics in PAH patients.

Study	n	Effusion size on echocardiography	Effusion size, qualitative	PC approach	Echo-guided PC	Real-time invasive hemodynamic monitoring during PC	Fluid description	Drainage (ml)	Drainage strategy
Ansari et al. (30)	1	25	Very large	Subxiphoid	—	—	—	2,400	400 ml removed on the first day and 2 L on subsequent days using a chest tube
Chen et al. (31)	1	50	—	—	—	—	Bloody	80	80 ml removed
Laimoud et al. (27)	1	26	Large	—	1	—	Serous	1,550	250 ml removed and gradual withdrawal of 1.3 L over the next 24 h
Yo et al. (32)	1	—	Large	—	—	—	Serosanguinous	1,000	1 L drained over 24 h
Fenstad et al. (33)	1	19	Large	—	1	—	—	800	800 ml removed over 3 days until drainage was <50 ml in 24 h
Fenstad et al. (13)	14	29 ± 9	Large (100%)	—	14 (100%)	—	Serous	750 (range, 350–2,200 ml)	Drainage over 2.9 ± 1 day
Singh et al. (14)	1	—	Large	Subxiphoid	1	1	Serosanguinous	320	Serial, low-volume PC over several days until fall in pericardial pressure below that of the RA and PA diastolic pressure
Hemnes et al. (17)	4	—	Large (100%)	—	—	—	—	566.67 ± 57.7	—
Batal et al. (34)	2	—	Large (100%)	—	—	—	—	1,300 ± 890.95	Patient 1: 670 ml removed Patient 2: 1,100 ml removed on the first day and 830 ml removed two days later
Case et al. (16)	3	—	Large (100%)	25% Apical 75% Subxiphoid	3 (100%)	—	—	616.67 ± 361.71	—
Weaver et al. (35)	1	—	Large	Subxiphoid	—	1	Serous	1,100	Gradual draining of 300 cc aliquots guided by the Swan-Ganz and the pericardial pressure monitoring with serial pressures and cardiac output measurements
Ruopp et al. (18)	1	—	Large	Subxiphoid	1	1	Serous	1,150	Gradual drainage of 200-ml aliquots until the pericardial pressure was less than both left- and right-sided diastolic pressures
Ruge et al. (36)	1	—	—	—	—	—	—	—	Placement of an indwelling pericardial catheter
Worsham et al. (28)	1	—	Moderate	—	—	—	Serous	—	—
Frey (37)	1	12	Large	—	—	—	—	350	350 ml removed

—, not reported; PC, pericardiocentesis.

**Table 4:** Hemodynamic data in PAH patients pre and post-pericardiocentesis.

Study	n	Right atrial pressure (mmHg)		Right ventricular end-diastolic pressure (mmHg)		Mean pulmonary artery pressure (mmHg)		Pulmonary capillary wedge pressure (mmHg)		Pulmonary vascular resistance (WU)		Cardiac index (L/min/m2)		Intrapericardial pressure (mmHg)	
		Pre	Post	Pre	Post	Pre	Post	Pre	Post	Pre	Post	Pre	Post	Pre	Post
Chen et al. (31)	1	—	—	—	—	75	—	36	—	4	—	5	—	—	—
Yo et al. (32)	1	—	6	—	—	—	35	—	11	—	8.7	—	1.6	—	—
Fenstad et al. (33)	1	—	—	—	—	78	—	—	—	13.9	—	2.14	—	—	—
Singh et al. (14)	1	28	17	28	—	48	50	24	39	8.6	2.6	1.5	2.2	25	10
Weaver et al. (35)	1	22	5	22	—	—	42	25	15	—	3.6	—	4.3	20	3
Ruopp et al. (18)	1	20	7	25	5	43	30	20	7	6.375	5.2	2.58	3.29	23	5
Ruge et al. (36)	1	—	6	—	11	—	41	—	12	—	4.8	—	3.32	—	—
Worsham et al. (28)	1	13	—	—	—	63	—	10	—	—	—	—	—	—	—
Frey (37)	1	21	21	28	—	—		—		—		—		12	<0

—, not reported; WU, wood units.

**Table 5:** 30-day peri-procedural complications and all-cause mortality.

Study	n	Major bleeding	Iatrogenic complication	Hemodynamic instability	Vasopressor requirement	CPR	PDS	30-day all-cause mortality	Other
Frantz et al. (29)	1	0	0	0	0	0	0	0	—
Ansari et al. (30)	1	0	0	0	1	0	0	1	—
Chen et al. (31)	1	0	0	1	0	1	0	1	—
Laimoud et al. (27)	1	0	0	1	1	1	1	1	VA—ECMO + Brain Death
Yo et al. (32)	1	0	0	0	0	0	0	0	—
Fenstad et al. (33)	1	0	0	0	0	0	0	0	—
Fenstad et al. (13)	14	0	0	0	0	0	0	2	—
Singh (14)	1	0	0	0	0	0	0	0	—
Hemnes et al. (17)	4	0	0	1	0	0	0	2	—
Batal et al. (34)	2	0	0	1	1	0	0	0	—
Case et al. (16)	3	0	0	0	0	0	0	0	—
Weaver et al. (35)	1	0	0	0	0	0	0	0	—
Ruopp et al. (18)	1	0	0	0	0	0	0	0	Repeat PC
Ruge et al. (36)	1	0	0	0	0	0	0	0	—
Worsham et al. (28)	1	0	0	0	0	0	0	0	—
Frey (37)	1	0	0	0	0	0	0	0	—

—, not reported; CPR, cardiopulmonary resuscitation; PDS, pericardial decompression syndrome; VA-ECMO, veno-arterial extracorporeal membrane oxygenation; PC, pericardiocentesis.

The echocardiographic characteristics reported in patients with PAH and large PE include moderate or more significant RV enlargement and dysfunction, likely related to the underlying PAH. Additionally, studies reported RA collapse (13, 18, 32, 36), RV collapse (14, 18, 32), left atrial (LA) collapse (13, 30, 33), left ventricular (LV) collapse (17, 28, 30, 37), diastolic hepatic vein flow reversal (13, 14, 33), >25% variation in mitral inflow (13, 14, 17, 33), interventricular septum shift (18, 27, 32, 33, 37), and inferior vena cava (IVC) plethora (30) (Table 6).

**Table 6:** Echocardiographic findings in patients with PAH and cardiac tamponade.

Study	Moderate or greater RA or RV enlargement	Moderate or greater RV dysfunction	RA or RV collapse	LA or LV collapse	Diastolic hepatic vein flow reversal	>25% Variation in mitral inflow	Shifting septum	IVC plethora	LVEF
Ansari et al. (30)	1	1	—	1	—	—	—	1	55
Chen et al. (31)	1	0	—	—	—	—	—	—	75
Laimoud et al. (27)	1	1	—	0	—	—	1	0	70
Yoet al. (32)	1	0	1	—	—	—	—	—	—
Fenstad et al. (33)	1	—	0	1	1	1	1	—	—
Fenstad et al. (13)	—	0	5	8	9	14	—	—	—
Singh et al. (14)	1	1	1	—	1	1	—	—	—
Hemnes et al. (17)	4	—	—	1	—	1	—	—	—
Batal et al. (34)	—	—	—	—	—	—	—	—	—
Case et al. (16)	1	1	—	—	—	—	—	—	55 ± 18.03
Ruopp et al. (18)	—	—	1	—	—	—	1	—	—
Ruge et al. (36)	1	1	1	—	—	—	—	—	75
Worsham et al. (28)	—	—	—	1	—	—	—	—	—
Frey (37)	1	1	0	1	—	—	1	—	—

—, not reported; RA, right atrial; RV, right ventricular; LA, left atrial; LV, left ventricular; IVC, inferior vena cava; LVEF, left ventricular ejection fraction.

## Discussion

This is the first systematic review that examines PC safety in patients with PAH presenting with large or hemodynamically significant PE. Our findings are as follows: (1) the most common phenotype consisted of young female patients with CTD-associated PAH; (2) the compiled mortality was 20% (7 of 35 patients), lower than what has been previously reported for PAH patients receiving PC; (3) post-procedure hemodynamic instability, including PDS, was the most frequent periprocedural complication across studies; (4) PC strategies varied, with most patients undergoing gradual drainage over hours or days; (5) most patients presented acutely with dyspnea and peripheral edema; and (6) the most common echocardiographic finding was left-sided chamber diastolic collapse, as opposed to the right-chamber collapse usually observed in cardiac tamponade.

Data regarding mortality rates associated with PC in patients with PAH is controversial, and its safety remains uncertain. Our study observed a 20% overall mortality rate following PC, with a range of 0% to 100%. Similarly, retrospective data from the National Inpatient Sample Database found a 25% unadjusted mortality rate in patients with PAH undergoing PC, which remained elevated after adjusting for confounders (38). Likewise, previous evidence has reported high post-PC mortality (50%) in patients with PAH (12, 17), supporting the use of alternative and more conservative treatment strategies. Conversely, several case reports have demonstrated favorable outcomes following PC in PAH when drainage is performed gradually (10, 14, 33). Differences in mortality rates may be partly explained by clinical heterogeneity within studies, PAH severity, underlying patient comorbidities, effusion size, drainage method, and timing of intervention. In contrast, among non-PAH populations, the complication rates associated with PC are significantly lower, ranging from 4% to 10%, depending on the clinical setting, type of monitoring, and operator skills (39).

Nevertheless, studies examining the outcomes of PC among patients with PAH are limited and do not provide definitive conclusions. Despite these discrepancies, the mortality rate associated with PC in patients with PAH is approximately four times higher than that observed in the general population (40–44). This observation may arise from the diagnostic challenges encountered in this population, where the absence of typical clinical and imaging findings often results in delayed treatment (13, 15, 45). Furthermore, the underlying heart disease may predispose these patients to an increased risk of complications (16, 46), such as PDS, as shown in our study. Additionally, it is essential to note that PE alone is an independent marker of mortality in patients with PAH (2, 10–13). These findings emphasize the importance of careful patient selection and risk assessment in patients with PAH undergoing PC.

Acute hemodynamic deterioration and/or pulmonary edema following PC characterizes PDS, although its definition remains inconsistent and lacks uniform application in clinical settings. Our study identified PDS in 14% of the patients, which differs from the reported general incidence of <5% (47). This discrepancy may be attributed to the underrecognition of the syndrome in the current literature. Nonetheless, studies involving patients with PAH have shown concerning mortality rates, potentially reflecting a less effective hemodynamic response in these patients (17). However, the exact mechanism by which PDS affects patients with PAH is unclear. Vandyke et al. (48) suggested that the sudden return of venous circulation and rapid re-expansion of the right chambers may lead to acute left-sided heart failure as a result of ventricular interdependence.

Additionally, a study evaluating RV changes following PC in patients with and without PH found no improvement in RV function in the PH group, in contrast to the enhanced function observed in patients without PH (49). In this context, RV decompression can lead to massive volume overload, which cannot be offset because of the persistently impaired ventricular function in these patients. An alternative hypothesis is that overdistention of the RV after PC may reduce coronary perfusion, causing RV ischemia. Furthermore, it has been proposed that large PE predominantly occurs in end-stage severe right heart failure in advanced PAH, where the right heart is unable to withstand additional stress due to its already deteriorated condition, even after PC. Further studies are needed to better understand the mechanisms underlying PDS in patients with PAH.

Case reports have recommended gradual decompression during PC in the presence of significant PH to prevent PDS (10, 14, 33). Echocardiography guidance during PC enables the identification of the proper site and the active monitoring of RV dynamics and cardiac output, allowing for the prompt delivery of hemodynamic support if needed. However, only 37.5% of the included studies reported echocardiography-guided PC. Moreover, real-time hemodynamic monitoring during PC allows drainage to continue until the pericardial pressure is below the bi-atrial diastolic pressures while preserving RV structural support to prevent an acute decompensation (18). It also enables the rapid identification of hemodynamic decline and informs the use of vasoactive therapies and more advanced interventions. The included studies that performed PC with a real-time Swan-Ganz and an intrapericardial pressure catheter reported no periprocedural complications. However, although we identified an overall improvement in cardiac hemodynamics after PC, more evidence is needed to identify a possible correlation. Recent evidence suggests that patients with PAH and large PE should undergo a right heart catheterization to evaluate hemodynamics before a pericardial procedure is contemplated (12, 50). Conversely, others propose hemodynamic monitoring immediately after PC for the early identification of impaired tissue perfusion and the need for cardiopulmonary support (27). Additionally, several studies suggest that the immediate use or initiation of PAH-specific therapy may contribute to successful post-drainage outcomes (4, 12). Although our findings suggest that hemodynamic monitoring may improve outcomes, it is essential to emphasize that these data are limited and somewhat anecdotal. This highlights the need for controlled studies that can support this proposal.

Cardiac tamponade typically presents a distinctive clinical presentation characterized by Beck's triad: hypotension due to reduced cardiac output, muffled heart sounds from PE, and jugular venous distention due to impaired venous return (51). In patients with PAH, the presentation of tamponade may differ. Adrian et al. (15) suggest that PAH may initially be a protective factor against cardiac tamponade. The increased pressures in the pulmonary system and RV enable the pericardium to hold a low fluid volume without causing right-chamber collapse. This results in a delayed presentation and rapid progression of symptoms that severely impact cardiac output once the protective effect wanes. Common signs and symptoms include dyspnea, tachycardia, peripheral edema, altered consciousness, and oxygen desaturation (52). Pulsus paradoxus is rarer because of the incapacity of the noncompliant RV to alter its filling volumes in response to the respiratory cycle. Hypotension may also be absent because of the compensatory increase in systemic vascular resistance (11). In our review, dyspnea and peripheral edema were the most frequently reported symptoms. However, these are nonspecific and are often misdiagnosed as right heart failure. As both conditions can present with similar clinical features, cardiac tamponade should be considered in the differential diagnosis of patients with right heart overload symptoms, and a bedside echocardiogram would be essential to rule out the possibility of PE. Early diagnosis is crucial in ensuring timely intervention and improving patient outcomes.

Typical echocardiographic signs of tamponade may be absent in the PAH population because of the increased right-sided pressures that “protect” against the rising pericardial pressure and maintain the RV output (53), concealing the classic presentation of cardiac tamponade. Limited evidence suggests that cardiac tamponade may occur in the absence of right-sided collapse and instead present with isolated effects on left-sided chambers. Signs like systolic RA collapse, RV diastolic collapse, IVC plethora, and reductions in blood flow velocities >25% across the mitral valve during inspiration are more infrequent. Accordingly, LV diastolic collapse occurred in several patients in our review, which is highly unusual and usually only develops in patients with loculated PE or severe PH (54). We also documented LA collapse and >25% variation in mitral flow more often than RA collapse and variation in tricuspid flow, respectively, suggesting a more evident effect of PE on the left heart. In a previous systematic review (15), 82% of patients with PAH had at least one unusual echocardiographic tamponade sign (IVC plethora, increased transvalvular respiratory variation, LA collapse, and LV collapse), and only 10.5% had both RA and RV collapse. This suggests that an echocardiographic evaluation in patients with PAH and a large PE should include a thorough assessment of the LA and LV for early echocardiographic tamponade identification.

Our systematic review has several significant limitations. This retrospective series of reported cases in the current literature has inherent selection and information biases. We included only articles that diagnosed PAH through right-heart catheterization, excluding several studies based on echocardiography diagnoses. While this approach ensured diagnostic accuracy and consistency across studies, it inevitably reduced the overall sample size. As a result, our findings should be interpreted with caution, as the limited number of cases restricts the generalizability of our conclusions. Furthermore, most of the included studies were case reports or small case series, which increases the potential for reporting bias, along with the inclusion of grey literature. The studies exhibited significant heterogeneity due to variations in study design, patient populations, and PC techniques, which complicated the generalization of findings and may have influenced our conclusions. Moreover, the broad definition of PDS may have further contributed to this variability, potentially leading to an overestimation of our findings and limiting comparability across studies. In addition, complete data could not be retrieved from all studies. Some of the conclusions drawn, notably the suggested association between hemodynamic monitoring and improved clinical outcomes, were not clearly supported by specific evidence; prospective, controlled data are needed. The absence of robust data underpinning this claim weakens the overall strength of the clinical recommendations derived from the literature. Also, there was ambiguity regarding the use and reporting of echocardiographic guidance. While echocardiography is regarded as essential in guiding pericardial interventions, only 37% of the studies explicitly reported its use. Whether the remaining studies omitted this technique or failed to report its utilization remains unclear. This inconsistency complicates the interpretation of outcomes and prevents definitive conclusions about the role of echocardiographic guidance in patient management. The heterogeneity among patients with connective tissue disease was not adequately addressed. Specifically, the studies did not differentiate the management strategy between inflammatory pericardial effusions and those associated with advanced right heart failure, two distinct clinical entities with potentially different therapies and prognoses. The lack of subgroup analysis limits the applicability of the findings to these diverse patient populations. Most included studies lacked control groups. This means that causal inferences should be drawn with caution. Finally, as with many systematic reviews, variations in study design, reporting quality, and potential publication bias across the included studies may have influenced the results. These limitations should be considered when interpreting the findings of this review Causal inferences should be drawn with caution.

A significant strength of this systematic review is that it is the first to provide evidence on the safety of PC in patients with PAH associated with tamponade or significant PE. We also employed a comprehensive and methodical approach to evidence collection and data extraction, utilizing multiple major databases and grey literature sources, alongside independent dual-reviewer screening and data extraction processes. Including studies across diverse designs (retrospective cohorts, case series, and case reports) provided a broad perspective on clinical presentations, management strategies, and outcomes of PC in patients with PAH and PE. Additionally, we contacted authors directly to confirm the diagnosis of PAH using right heart catheterization when it was unclear. We included case reports to capture real-world scenarios more effectively and improve the generalizability of our study, which we consider appropriate given the rarity of the studied condition. Despite the overall low quality of the included studies, the review's systematic and exhaustive nature allows for valuable insights into procedural approaches, hemodynamic changes, and potential complications, such as PDS.

Our findings suggest that PC in the setting of large or hemodynamically significant PE in patients with PAH is linked to a high mortality rate (20%) and PDS, particularly among young women with CTD-associated PAH. However, these rates are lower than those previously reported. The clinical presentation often mimics right heart failure, and typical imaging findings may be absent. Therefore, clinicians should prioritize evaluating LV and LA diastolic collapse in the presence of significant PE in patients with PAH. Echocardiography-guided and gradual decompression during PC, with or without real-time hemodynamic monitoring, shows potential for improved outcomes, although further research is needed to confirm these findings.

## Data Availability

The original contributions presented in the study are included in the article/Supplementary Material, further inquiries can be directed to the corresponding author.
