# Supplementary material for: Safety of pericardiocentesis in pulmonary arterial hypertension: a systematic review
Source: Front Cardiovasc Med. 2025 Aug 18;12:1610419. doi: 10.3389/fcvm.2025.1610419 (PMC12400511; doi:10.3389/fcvm.2025.1610419)
Supplement: Supplementary file 1 [file Datasheet1.docx]

**Supplemental material**

Table of Contents

Supplemental material 1. Appendix S1 3

Supplemental material 2. Appendix S2 4

Supplemental material 3. Appendix S3 5

Supplemental material 4. Appendix S4 9

Supplemental material 5. Table S1 11

Supplemental material 6. Table S2 13

Supplemental material 7. Table S3 15

Supplemental material 8. Table S4 16

Supplemental material 9. Table S5 18

Supplemental material 10. Table 6 19

Supplemental material 11. Table S7 21

Supplemental material 12. Table S8 22

Supplemental material 1. Appendix S1

*Search strategy*

PubMed

- (pulmonary arterial hypertension[MeSH Terms]) AND (pericardiocentesis[MeSH Terms])

Google Scholar

- "pulmonary arterial hypertension" AND "pericardial effusion" AND pericardiocentesis

Scopus

- "pulmonary arterial hypertension" AND "pericardial effusion" AND “pericardiocentesis”

Web of Science

- ((pulmonary arterial hypertension[Topic]) AND (pericardial effusion[Topic])) AND (pericardiocentesis[Topic])

OpenGrey

- "pulmonary arterial hypertension" AND "pericardial effusion" AND pericardiocentesis

Supplemental material 2. Appendix S2

*MeSH terms*

- Pulmonary arterial hypertension
- Group 1 pulmonary hypertension
- Pericardial effusion
- Pericardiocentesis
- Pericardial drainage
- Pericardial effusion drainage
- Cardiac tamponade
- Pericardial tamponade
- Pericardial decompression syndrome
- Safety
- Effectiveness
- Mortality
- Iatrogenic complication
- Bleeding
- Hemodynamic compromise
- Circulatory collapse
- Cardiopulmonary resuscitation
- Postoperative low cardiac output syndrome
- Paradoxical hemodynamic instability

Supplemental material 3. Appendix S3

*Controlled vocabulary*

- *Pulmonary arterial hypertension (PAH):* mean pulmonary arterial pressure (mPAP) ≥20 mmHg at rest, measured by right heart catheterization, pulmonary artery wedge pressure ≤15 mmHg, and pulmonary vascular resistance > 2 Wood units (1).
- Pericardial effusion: Abnormal accumulation of pericardial fluid in the pericardial cavity (2).
- *Trivial:* seen only in systole, corresponding to <50 mL.
- *Small:* <10 mm, corresponding to 50-100 mL pericardial fluid.
- *Moderate:* 10-20 mm, corresponding to 100-500 mL pericardial fluid.
- *Large:* >20 mm, corresponding to >500 mL pericardial fluid.
- *Very large:* >25 mm, corresponding to >700 mL pericardial fluid.
- *Cardiac tamponade:* cardiac compression due to fluid accumulation within the pericardial sac, resulting in impaired diastolic filling of the ventricles (3).
- *Clinical diagnosis:* dyspnea, jugular venous distention, pulsus paradoxus, hypotension, and muffled heart sounds (4), elevated systemic venous pressure, pulsus paradoxus, dyspnea, and tachycardia (3).
- *Echocardiographic diagnosis:* specific features observed on echocardiography that suggest the presence of this condition, with each feature having variable sensitivity and specificity.
- *Pericardial effusion:* A large pericardial effusion with a swinging heart is suggestive of cardiac tamponade (3).
- *Diastolic collapse of the right ventricle*: Inversion of the right ventricular wall during early diastole, when intracavitary right ventricular pressure and volume are at their lowest, is a sign of cardiac tamponade. This finding is typically observed in the parasternal long-axis view on echocardiography, though it is uncommon in patients with PAH.
- *Diastolic collapse of the right atrium*: Inversion of the right atrial wall, beginning in late diastole, is an early echocardiographic sign of cardiac tamponade. It becomes more specific if the collapse persists for more than 30% of the cardiac cycle, though this finding is uncommon in patients with PAH.
- *Diastolic collapse of the left ventricle*: LV collapse typically occurs in early diastole and is localized to the posterior or lateral free wall. While this finding rarely occurs with circumferential, non-loculated pericardial effusions, it is more common in these types of pericardial effusions in patients with severe pulmonary arterial hypertension *(5,6).*
- *Diastolic collapse of the left atrium:* usually seen in up to 25% of general cardiac tamponade cases, and it may be the only sign of cardiac tamponade in patients with PAH alongside left ventricular collapse due to increased right-sided pressures that prevent the right ventricle from collapsing (7,8).
- *Inferior vena cava plethora:* This condition is characterized by a dilated inferior vena cava (IVC) with a diameter greater than 20 mm and less than a 50% reduction in size during respiratory phases. It suggests elevated intra-pericardial pressure transmitted to the right heart chambers and is highly sensitive to the physiology of cardiac tamponade, including in patients with PAH.
- *Doppler signs of increased ventricular interdependence: S*een in cases of cardiac tamponade, where increasing pericardial fluid leads cardiac chambers competing for space. The Doppler signs include:
- *Increased mitral inflow E velocity:* Refers to the peak velocity of blood flow through the mitral valve during the early phase of diastole, when the left ventricle is filling with blood. A decrease of more than 25% in the E wave velocity of the mitral inflow during inspiration compared to expiration indicates significant tamponade physiology (9).
- *Increased tricuspid inflow E velocity:* Refers to the peak velocity of blood flow through the tricuspid valve during the early phase of diastole, when the right ventricle is filling with blood. A decrease of more than 40% in the tricuspid inflow E velocity inflow during inspiration compared to expiration is considered consistent with significant tamponade physiology (9).
- *Tricuspid Annular Plane Systolic Excursion (TAPSE):* TAPSE measures the longitudinal function of the right ventricle, reflecting its systolic performance.
- *Pericardiocentesis*: a medical procedure in which a needle is inserted into the pericardial sac to aspirate excess fluid or blood. This intervention is typically performed to relieve cardiac tamponade symptoms or obtain diagnostic samples from the pericardial space *(3)*.
- *Mayor periprocedural complication:* defined as death, major bleeding, traumatic injury to cardiac structures, injury to surrounding structures, significant hemodynamic compromise, or circulatory collapse requiring cardiopulmonary resuscitation, intraprocedurally or up to 30 days post-procedure (10).
- *Death:* permanent cessation of all vital functions, including heartbeat, respiration, and brain activity. It represents the end of life and is often confirmed through clinical criteria such as the irreversible loss of circulatory and respiratory functions or the diagnosis of brain death (11)
- *In-hospital mortality:* death occurring within the hospital during the admission period of a patient.
- *Major bleeding:* fatal bleeding; and/or symptomatic bleeding in a critical area or organ, such as intracranial, intraspinal, intraocular, retroperitoneal, intra-articular or pericardial, or intramuscular with compartment syndrome; and/or bleeding causing a fall in hemoglobin level of 20 g L^−1^ (1.24 mmol L^−1^) or more, or leading to transfusion of two or more units of whole blood or red cells (12).
- *Traumatic myocardial injury:* damage to the heart muscle resulting from blunt or penetrating trauma *(13).*
- *Hemodynamic instability*: a substantial and detrimental alteration in the balance of blood flow and pressure within the cardiovascular system, leading to impaired organ perfusion and function. This condition is characterized by severe deviations from normal blood pressure, cardiac output, or blood volume, resulting in clinical manifestations such as hypotension, shock, or organ dysfunction *(14).*
- *Hypotension:* a condition characterized by abnormally low blood pressure, which can result in symptoms such as dizziness, fainting, and blurred vision. It may be a sign of an underlying health issue or a reaction to medications or other interventions (15)*.*
- *Pericardial decompression syndrome:* a life-threatening complication that may arise following the evacuation of fluid from the pericardial sac, characterized by the rapid re-expansion of the heart and the sudden increase in venous return, which can lead to acute left ventricular dysfunction, pulmonary edema, and hemodynamic instability (16). Various terminologies have been designated for this complication, including Paradoxical Hemodynamic Instability (PHI) and postoperative low cardiac output syndrome (POLCOS) (17–19).
- *Shock:* a critical condition characterized by a significant reduction in blood flow and perfusion to vital organs, leading to inadequate oxygen delivery and potential organ failure. It can result from various causes, including hypovolemia, cardiogenic dysfunction, or septic infection (20).
- *Circulatory collapse requiring cardiopulmonary resuscitation:* a critical condition where there is a sudden and severe failure of the circulatory system, resulting in the cessation of effective blood flow and oxygen delivery to vital organs, in which immediate cardiopulmonary resuscitation is required to restore circulation and respiratory function, which involves chest compressions, artificial ventilation, and potentially the use of defibrillation or medications to reestablish normal heart rhythm and hemodynamics*.*
- *Vasopressor requirement:* need for pharmacological agents that induce vasoconstriction to elevate blood pressure in patients experiencing hypotension (21).
- Need for mechanical circulatory support: the requirement for devices or technologies to assist or replace failing bodily functions, such as cardiac pumps or ventilators (22).

*References*

1. Humbert M, Kovacs G, Hoeper MM, Badagliacca R, Berger RMF, Brida M, et al. 2022 ESC/ERS Guidelines for the diagnosis and treatment of pulmonary hypertension. European Heart Journal. 2022 Oct 11;43(38):3618–731.

2. Weitzman LB, Tinker WP, Kronzon I, Cohen ML, Glassman E, Spencer FC. The incidence and natural history of pericardial effusion after cardiac surgery--an echocardiographic study. Circulation. 1984 Mar;69(3):506–11.

3. Fowler NO. Cardiac tamponade. A clinical or an echocardiographic diagnosis? Circulation. 1993 May;87(5):1738–41.

4. Zipes DP, Libby P, Bonow RO, Mann DL, Tomaselli GF, Braunwald E, editors. Braunwald’s heart disease: a textbook of cardiovascular medicine. Eleventh edition, international edition. Philadelphia, PA: Elsevier; 2019. 1944 p.

5. Kuvin JT, Khabbaz K, Pandian NG. Left Ventricular Apical Diastolic Collapse: An Unusual Echocardiographic Marker of Postoperative Cardiac Tamponade. Journal of the American Society of Echocardiography. 1999 Mar;12(3):218–20.

6. Kumar B, Kodliwadmath A, Singh A, Upadhyay A, Darbari A, Duggal B. Left ventricular tamponade- pathophysiology determines the therapeutic approach: a case series. Dastidar AG, Brown RA, Mehta OH, Kurdi H, Jakstaite AM, editors. European Heart Journal - Case Reports. 2021 Feb 4;5(2):ytaa502.

7. Adams JR, Tonelli AR, Rokadia HK, Duggal A. Cardiac Tamponade in Severe Pulmonary Hypertension. A Therapeutic Challenge Revisited. Annals ATS. 2015 Mar;12(3):455–60.

8. Ansari Aval Z, Mirhosseini SM, Jafari Naeini S. Atypical presentation of cardiac tamponade in pulmonary hypertension: A case report and review of the literature. Clinical Case Reports. 2021 Dec;9(12):e05218.

9. Burstow DJ, Oh JK, Bailey KR, Seward JB, Tajik AJ. Cardiac Tamponade: Characteristic Doppler Observations. Mayo Clinic Proceedings. 1989 Mar;64(3):312–24.

10. Case BC, Yang M, Kagan CM, Yerasi C, Forrestal BJ, Tariq MU, et al. Safety and Feasibility of Performing Pericardiocentesis on Patients with Significant Pulmonary Hypertension. Cardiovascular Revascularization Medicine. 2019 Dec;20(12):1090–5.

11. Death’s Troubled Relationship With the Law. AMA Journal of Ethics. 2020 Dec 1;22(12):E1055-1061.

12. Schulman S, Kearon C. Definition of major bleeding in clinical investigations of antihemostatic medicinal products in non‐surgical patients. Journal of Thrombosis and Haemostasis. 2005 Apr;3(4):692–4.

13. Eiferman D, Nathan R, Firstenberg M. Cardiac Trauma. In: Firstenberg M, editor. Principles and Practice of Cardiothoracic Surgery [Internet]. InTech; 2013 [cited 2024 Aug 12]. Available from: http://www.intechopen.com/books/principles-and-practice-of-cardiothoracic-surgery/cardiac-trauma

14. Weil MH. Defining Hemodynamic Instability. In: Pinsky MR, Payen D, editors. Functional Hemodynamic Monitoring [Internet]. Berlin/Heidelberg: Springer-Verlag; 2005 [cited 2024 Aug 12]. p. 9–17. (Update in Intensive Care and Emergency Medicine; vol. 42). Available from: http://link.springer.com/10.1007/3-540-26900-2_2

15. Brady KM, Hudson A, Hood R, DeCaria B, Lewis C, Hogue CW. Personalizing the Definition of Hypotension to Protect the Brain. Anesthesiology. 2020 Jan 1;132(1):170–9.

16. Sobieski C, Herner M, Goyal N, Khor LL, Chang L, Bieging E, et al. Pericardial Decompression Syndrome After Drainage of Chronic Pericardial Effusions. JACC: Case Reports. 2022 Nov;4(22):1515–21.

17. Dosios T, Theakos N, Angouras D, Asimacopoulos P. Risk Factors Affecting the Survival of Patients With Pericardial Effusion Submitted to Subxiphoid Pericardiostomy. Chest. 2003 Jul;124(1):242–6.

18. Amro A, Mansoor K, Amro M, Sobeih A, Suliman M, Okoro K, et al. A Comprehensive Systemic Literature Review of Pericardial Decompression Syndrome: Often Unrecognized and Potentially Fatal Syndrome. CCR. 2021 Jan;17(1):101–10.

19. Han AJ, Slomka T, Mehrotra A, Murillo LC, Alsafwah SF, Khouzam RN. Paradoxical Hemodynamic Instability After Pericardial Window. Echocardiography. 2016 Aug;33(8):1251–2.

20. Standl T, Annecke T, Cascorbi I, Heller AR, Sabashnikov A, Teske W. The Nomenclature, Definition and Distinction of Types of Shock. Deutsches Ärzteblatt international [Internet]. 2018 Nov 9 [cited 2024 Aug 12]; Available from: https://www.aerzteblatt.de/10.3238/arztebl.2018.0757

21. Bloom JE, Chan W, Kaye DM, Stub D. State of Shock: Contemporary Vasopressor and Inotrope Use in Cardiogenic Shock. JAHA. 2023 Aug;12(15):e029787.

22. Tam CW, Shen L, Zeidman AD, Srivastava A, Ivascu NS. Mechanical Circulatory Support: Primer for Consultant Specialists. CJASN. 2022 Jun;17(6):890–901.

Supplemental material 4. Appendix S4

*Variables of interest for data extraction*

Study characteristics

- Type of study
- DOI
- Journal
- Country
- Year of publication
- Author
- Title

Baseline patient characteristics

- Number of patients
- Age
- Female
- BMI
- PAH etiology
- Time since PAH diagnosis
- PAH therapy
- Comorbidities
- WHO functional class

Clinical presentation

- Heart rate
- Systolic blood pressure
- Diastolic blood pressure
- Vasopressors
- Respiratory rate
- O2 saturation
- Peripheral edema
- Presyncope
- Syncope
- Palpitations
- Shock
- Altered mental status
- Dyspnea
- Chest pain
- Fatigue
- Loud S2
- Orthopnea
- Faint or absent heart sounds
- Jugular venous distention
- Pulsus paradoxus

Echocardiographic findings

- Moderate or greater right atrial enlargement
- Moderate or greater right ventricular enlargement
- Moderate or greater right ventricular dysfunction
- Estimated right atrial pressure
- Right atrial systolic collapse
- Right ventricular diastolic collapse
- Left atrial systolic collapse
- Left ventricular diastolic collapse
- IVC plethora
- Diastolic hepatic vein flow reversal
- >25% variation in mitral inflow
- >40% variation in tricuspid inflow
- Septal shift
- Estimated right ventricular systolic pressure
- Pulmonary artery systolic pressure
- TAPSE
- Tamponade physiology
- Left ventricular ejection fraction

Right heart catheterization

- Right atrial pressure
- Central venous pressure
- Mean pulmonary artery pressure
- Left ventricular end-diastolic pressure
- Right ventricular end-diastolic pressure
- Pulmonary capillary wedge pressure
- Cardiac index
- Pulmonary vascular resistance
- Pulmonary artery pulsatility index
- Systemic vascular resistance
- Intra-pericardial pressure

Pericardial effusion and pericardiocentesis data

- Etiology
- Effusion size, quantitative
- Effusion size, qualitative
- Fluid description
- Pericardiocentesis approach
- Echo-guided pericardiocentesis
- Real-time invasive hemodynamic monitoring during pericardiocentesis
- Total drainage
- Drainage strategy

Major periprocedural complications

- Major bleeding
- Iatrogenic complication
- Hemodynamic instability
- Shock
- Vasopressor requirement
- Circulatory collapse requiring CPR
- Pericardial decompression syndrome
- 30-day all-cause mortality

Supplemental material 5. Table S1

*Study characteristics*

| ID | Title | Author | Journal | Year of publication | Country | DOI | Type of study |
| --- | --- | --- | --- | --- | --- | --- | --- |
| 1 | Ambulatory Hemodynamic Monitoring in Pulmonary Arterial Hypertension | Frantz, R.P | Advances in Pulmonary Hypertension | 2008 | United States | 10.21693/1933-088X-7.4.405 | Prospective cohort |
| 2 | Atypical presentation of cardiac tamponade in pulmonary hypertension: A case report and review of the literature | Ansari, Z | Wiley | 2021 | Iran | 10.1002/ccr3.5218 | Case report |
| 3 | Cardiac Arrest After a Diagnostic Pericardiocentesis in a Patient With Severe Pulmonary Arterial Hypertension | Chen, E | American Journal of Respiratory and Critical Care Medicine | 2024 | United States | 10.1164/ajrccm-conference.2024.209.1_MeetingAbstracts.A2230 | Abstract |
| 4 | Emergency Veno-Arterial Extracorporeal Membrane Oxygenation for Pericardial Decompression Syndrome | Laimoud, M | Hindawi Case Reports in Cardiology | 2022 | Saudi Arabia | 10.1155/2022/5440635 | Case report |
| 5 | Incident Pulmonary Arterial Hypertension Associated with Bosutinib | Yo, S | Pulmonary Circulation | 2020 | Canada | 10.1177/2045894020936913 | Case report |
| 6 | Pericardial Effusion in a Patient with Pulmonary Arterial Hypertension | Fenstad, E.R | The Journal of Heart and Lung Transplantation | 2011 | United States | 10.1016/j.healun.2011.01.516 | Abstract |
| 7 | Pericardial Effusions in Pulmonary Arterial Hypertension: Characteristics, Prognosis, and Role of Drainage | Fenstad, E.R | Chest | 2013 | United States | 10.1378/chest.12-3033 | Retrospective cohort |
| 8 | Pericardiocentesis in Severe Pulmonary Arterial Hypertension Guided by a Pulmonary Artery Catheter | Singh, A | JACC: Case Reports | 2024 | United States | 10.1016/j.jaccas.2024.102339 | Case report |
| 9 | Poor Outcomes Associated with Drainage of Pericardial Effusions in Patients with Pulmonary Arterial Hypertension | Hemnes, A.R | Southern Medical Association Journal | 2007 | United States | 10.1097/SMJ.0b013e31816c0169 | Case series |
| 10 | Prognostic Value of Pericardial Effusion on Serial Echocardiograms in Pulmonary Arterial Hypertension | Batal, O | Echocardiography | 2015 | United States | 10.1111/echo.12909 | Retrospective cohort |
| 11 | Safety and Feasibility of Performing Pericardiocentesis on Patients with Significant Pulmonary Hypertension | Case, B | Cardiovascular Revascularization Medicine | 2019 | United States | 10.1016/j.carrev.2019.09.022 | Retrospective cohort |
| 12 | Swan-Ganz-and intra-pericardial pressure guided pericardiocentesis in scleroderma-associated PAH | Weaver, M | Pulmonary Vascular Disease | 2021 | United States | 10.1016/j.chest.2021.07.1949 | Abstract |
| 13 | Swan-Ganz and Pericardial Pressure–guided Pericardiocentesis in Pulmonary Arterial Hypertension–associated Cardiac Tamponade | Ruopp, N | Annals of the American Thoracic Society | 2019 | United States | 10.1513/AnnalsATS.201902-127CC | Case report |
| 14 | The Value of Right Heart Catheterization  Case Series Showing Benefits in a Variety of Diagnoses | Ruge, M | JACC: Case Reports | 2023 | United States | 10.1016/j.jaccas.2023.101959 | Case series |
| 15 | Worsening Dyspnea in a Patient with Connective Tissue Disease and Pulmonary Arterial Hypertension | Worsham, C.M | American Journal of Respiratory and Critical Care | 2017 | United States | 10.1164/ajrccm-conference.2017.195.1_MeetingAbstracts.A6195. | Abstract |
| 16 | Recognition of Cardiac Tamponade in the Presence of Severe Pulmonary Hypertension | Frey, M.J | Medicine Annals of Internal Medicine | 1989 | United States | 10.7326/0003-4819-111-7-615 | Case report |

Supplemental material 6. Table S2

*Baseline patient characteristics*

| ID | N | Age | Female (%) | PAH etiology | Time since PAH diagnosis (months) | PAH therapy (%) | Comorbidities | WHO functional class |
| --- | --- | --- | --- | --- | --- | --- | --- | --- |
| 1 | 1 | - | - | - | - | 100 | - | - |
| 2 | 1 | 32 | 100 | Associated with CTD (SSc) | 84 | 100 | - | - |
| 3 | 1 | 61 | 100 | Idiopathic | - | 100 | - | - |
| 4 | 1 | 28 | 100 | Idiopathic | 36 | 100 | - | - |
| 5 | 1 | 37 | 100 | Associated with drugs and toxins | 0 | 0 | - | 3 |
| 6 | 1 | 54 | 100 | Associated with CTD (limited scleroderma) | 0 | 0 | - | 4 |
| 7 | 14 | 54 ± 9 | 42.9 | 71% associated with CTD | - | - | - | - |
| 8 | 1 | 33 | 100 | Associated with CTD (RA and SSc overlap syndrome) | 5 | 100 | Diabetes mellitus, hypertension | 3 |
| 9 | 4 | 38.75 ± 3.5 | 75 | 25% Idiopathic  25% Associated with drugs and toxins  25% Associated with CTD (scleroderma)  25% Associated with portal hypertension | 38.5 ± 55.16 | 100 | - | 3.5 ± 0.58 |
| 10 | 2 | - | - | Associated with CTD (50% SSc, 50% scleroderma) | - | 50 | - | - |
| 11 | 3 | 60.67 ± 7.5 | 100 | 66.7% Associated with CTD (50% SLE, 50% RA) | - | - | - | - |
| 12 | 1 | 57 | 100 | Associated with CTD (scleroderma type 1) | 24 | 100 | - | - |
| 13 | 1 | 60 | 100 | Associated with HIV infection | 240 | 100 | - | - |
| 14 | 1 | 51 | 100 | Associated with CTD (antisynthetase syndrome and possible Sjogren’s syndrome) | 0 | 0 | - | - |
| 15 | 1 | 31 | 100 | Associated with CTD (SSc and SLE overlap syndrome) | - | 100 | - | - |
| 16 | 1 | 44 | 100 | Idiopathic | 12 | - | - | - |

-: not reported, CTD: connective tissue disease, SSc: systemic sclerosis, RA: rheumatoid arthritis, SLE: systemic lupus erythematosus

Supplemental material 7. Table S3

*Clinical presentation**

| ID | HR (bpm) | BP (mmHg) | Vasopressors (n) | RR (rpm) | O2 Sat (%) | Dyspnea (n) | Peripheral edema (n) | Presyncope (n) | Loud S2 (n) | Faint of absent heart sounds (n) | JVD (n) | Others (n) |
| --- | --- | --- | --- | --- | --- | --- | --- | --- | --- | --- | --- | --- |
| 1 | - | - | - | - | - | 1 | - | - | - | - | - | - |
| 2 | 120 | 80/55 | - | - | 75 | 1 | 1 | 0 | 0 | 1 | 1 | 0 |
| 3 | - | 93/39 | - | - | - | 1 | 1 | 0 | 0 | 0 | 0 | 0 |
| 4 | 130 | 90/45 | 1 | - | 80 | 1 | 1 | 0 | 0 | 0 | 1 | Palpitations (1) |
| 5 | - | Normotensive | - | - | - | 1 | - | - | - | - | - | - |
| 6 | - | - | - |  | - | 1 | 1 | 1 | 1 | 0 | 0 | 0 |
| 7 | 101 ± 12 | 106 ± 17 / 63 ± 12 | - | - | - | 14 (100%) | - | - | - | - | - | - |
| 8 | 150 | 74/48 | 1 | - | - | 1 | 1 | - | - | 1 | 1 | Chest pain (1) |
| 9 | - | - | 1 (25%) | - | - | - | - | - | - | - | - | - |
| 10 | - | - | - | - | - | - | - | - | - | - | - | - |
| 11 | - | - | - | - | - | - | - | - | - | - | - | - |
| 12 | - | - | - | - | - | 1 | 1 | - | - | - | - | - |
| 13 | - | 70/50 | - | - | - | - | 1 | - | - | - | - | - |
| 14 | 118 | - | - | - | 88 | 1 | 1 | - | - | - | 1 | Pulsus paradoxus (1) |
| 15 | - | - | - | - | - | 1 | - | 1 | - |  | - | Chest pain (1) |
| 16 | 90 | 120/60 | - | 18 | - | 1 | 1 | - | 1 | - | 1 | - |

-: not reported, HR: heart rate, BP: blood pressure, RR: respiratory rate, O2 Sat: O2 saturation, JVD: jugular venous distention

*Orthopnea, fatigue, and syncope are not reported in any of the included studies.

Supplemental material 8. Table S4

*Echocardiographic findings**

| **ID** | **Moderate or greater RA enlargement (n)** | **Moderate or greater RV enlargement (n)** | **Moderate or greater RV dysfunction (n)** | **RA collapse (n)** | **RV collapse (n)** | **LA collapse (n)** | **LV collapse (n)** | **Diastolic hepatic vein flow reversal (n)** | **>25% Variation in mitral inflow (n)** | **Shifting septum (n)** | **Estimated RVSP (mmHg)** | **sPAP (mmHg)** | **LVEF (%)** | **Others** |
| --- | --- | --- | --- | --- | --- | --- | --- | --- | --- | --- | --- | --- | --- | --- |
| 1 | - | - | - | - | - | - | - | - | - | - | - | - | - |  |
| 2 | - | 1 | 1 | - | 0 | 1 | 1 | - | - | - | - | 80 | 55 | IVC plethora: 1 |
| 3 | 1 | 1 | 0 | - | - | - | - | - | - | - | 135 | - | 75 |  |
| 4 | 1 | 1 | 1 | - | - | 0 | 0 | - | - | 1 | - | 115 | 70 |  |
| 5 | - | 1 | 0 | 1 | 1 | - | - | - | - | - | - | 55 | - |  |
| 6 | - | 1 | - | 0 | 0 | 1 | - | 1 | 1 | 1 | 98 | - | - |  |
| 7 | - | - | 0 | 5 (35.7%) | 0 | 8 (57.1%) | - | 9 (57.1%) | 14 | - | - | - | - | Estimated RA pressure: 16 ± 5 mmHg |
| 8 | - | 1 | 1 | - | 1 | 1 | - | 1 | 1 | - | - | - | - |  |
| 9 | 4 (100%) | 4 (100%) | - | - | - | - | 1 (25%) | - | 1 (25%) | - | - | - | - |  |
| 10 | - | - | - | - | - | - | - | - | - | - | - | - | - |  |
| 11 | - | 1 (33.3%) | 1 (33.3%) | - | - | - | - | - | - | - | - | - | 55 ± 18.03 |  |
| 12 | - | - | - | - | - | - | - | - | - | - | - | - | - |  |
| 13 | - | - | - | 1 | 1 | - | - | - | - | 1 | - | - | - |  |
| 14 | - | 1 | 1 | 1 | - | - | - | - | - | - | - | 66 | 75 |  |
| 15 | - | - | - | - | - | - | 1 | - | - | - | - | - | - |  |
| 16 | 1 | 1 | 1 | - | 0 | - | 1 | - | - | 1 | - | - | - |  |

-: not reported, RA: right atrial, RV: right ventricular, LA: left atrial, LV: left ventricular, IVC: inferior vena cava, RVSP: right ventricular systolic pressure, sPAP: systolic pulmonary artery pressure, LVEF: left ventricular ejection fraction

*TAPSE and >40% variation in tricuspid inflow are not reported in any of the included studies.

Supplemental material 9. Table S5

*Right heart catheterization pre- and post-pericardiocentesis*

| **ID** | **Central venous pressure (mmHg)** | | **Right atrial pressure (mmHg)** | | **Right ventricular end-diastolic pressure (mmHg)** | | **Mean pulmonary artery pressure (mmHg)** | | **Pulmonary capillary wedge pressure (mmHg)** | | **Pulmonary vascular resistance (WU)** | | **Systemic vascular resistance (dyn/s/cm^-5^)** | | **Cardiac index (L/min/m2)** | | **Intrapericardial pressure (mmHg)** | |
| --- | --- | --- | --- | --- | --- | --- | --- | --- | --- | --- | --- | --- | --- | --- | --- | --- | --- | --- |
|  | Pre | Post | Pre | Post | Pre | Post | Pre | Post | Pre | Post | Pre | Post | Pre | Post | Pre | Post | Pre | Post |
| 1 | - | - | - | - | - | - | - | - | - | - | - | - | - | - | - | - | - | - |
| 2 | - | - | - | - | - | - | - | - | - | - | - | - | - | - | - | - | - | - |
| 3 | 15 | - | - | - | - | - | 75 | - | 36 | - | 4 | - | 548 | - | 5 | - | - | - |
| 4 | 25 | - | - | - | - | - | - | - | - | - | - | - | - | - | - | - | - | - |
| 5 | - | - | - | 6 | - | - | - | 35 | - | 11 | - | 8.7 | - | - | - | 1.6 | - | - |
| 6 | - | - | - | - | - | - | 78 | - | - | - | 13.9 | - | - | - | 2.14 | - | - | - |
| 7 | - | - | - | - | - | - | - | - | - | - | - | - | - | - | - | - | - | - |
| 8 | - | - | 28 | 17 | 28 | - | 48 | 50 | 24 | 39 | 8.6 | 2.6 | - | - | 1.5 | 2.2 | 25 | 10 |
| 9 | - | - | - | - | - | - | - | - | - | - | - | - | - | - | - | - | - | - |
| 10 | - | - | - | - | - | - | - | - | - | - | - | - | - | - | - | - | - | - |
| 11 | - | - | - | - | - | - | 39 ± 13.75 | - | - | - | - | - | - | - | - | - | - | - |
| 12 | - | - | 22 | 5 | 22 | - | - | 42 | 25 | 15 | - | 3.6 | - | - | - | 4.3 | 20 | 3 |
| 13 | - | - | 20 | 7 | 25 | 5 | 43 | 30 | 20 | 7 | 6.375 | 5.2 | 1175 | 1284 | 2.58 | 3.29 | 23 | 5 |
| 14 | - | - | - | 6 | - | 11 | - | 41 | - | 12 | - | 4.8 | - | 2091 | - | 3.32 | - | - |
| 15 | - | - | 13 | - | - | - | 63 | - | 10 | - | - | - | - | - | - | - | - | - |
| 16 | 12 | - | 21 | 21 | 28 | - | - | - | - | - | - | - | - | - | - | - | 12 | <0 |

-: not reported

Supplemental material 10. Table S6

*Pericardial effusion and pericardiocentesis data*

| **ID** | **Effusion etiology** | **Effusuion size on echocardiography (mm)** | **Effusuion size, qualitative** | **PC approach** | **Echo- guided PC** | **Real-time invasive hemodynamic monitoring during PC** | **Fluid description** | **Drainage (mL)** | **Drainage strategy** |
| --- | --- | --- | --- | --- | --- | --- | --- | --- | --- |
| 1 | - | - | - | - | - | - | - | - | - |
| 2 | - | 25 | Very large | Subxiphoid | - | - | - | 2400 | 400 mL removed on the first day and 2 L on subsequent days using a chest tube |
| 3 | PAH* | 50 | - | - | - | - | Bloody | 80 | 80 mL removed |
| 4 | PAH* | 26 | Large | - | 1 | - | Serous | 1550 | 250 mL removed and gradual withdrawal of 1.3 L over the next 24 hours |
| 5 | PAH* | - | Large | - | - | - | Serosanginous | 1000 | 1 L drained over 24 hours |
| 6 | PAH* | 19 | Large | - | 1 | - | - | 800 | 800 mL removed over 3 days until drainage was <50 ml in 24 hours |
| 7 | Autoimmune disease | 29 ± 9 | Large (100%) | - | 14 (100%) | - | Serous | 750 (range, 350-2,200 mL) | Drainage over 2.9 ± 1 days |
| 8 | Autoimmune disease and/or PAH* | - | Large | Subxiphoid | 1 | 1 | Serosanginous | 320 | Serial, low-volume PC over several days until fall in pericardial pressure below that of the RA and PA diastolic pressure |
| 9 | PAH* | - | Large (100%) | - | - | - | - | 566.67 ± 57.7 | - |
| 10 | PAH* | - | Large (100%) | - | - | - | - | 1300 ± 890.95 | Patient 1: 670 mL removed  Patient 2: 1100 mL removed on first day and 830 mL removed two days later |
| 11 | PAH* | - | Large (100%) | 25% Apical  75% Subxiphoid | 3 (100%) | - | - | 616.67 ± 361.71 | - |
| 12 | 66.7% Autoimmune disease  33.3% PAH | - | Large | Subxiphoid | - | 1 | Serous | 1100 | Gradual draining of 300 cc aliquots guided by the Swan-Ganz and the pericardial pressure monitoring with serial pressures and cardiac output measurements |
| 13 | PAH* | - | Large | Subxiphoid | 1 | 1 | Serous | 1150 | Gradual drainage of 200-ml aliquots until the pericardial pressure was less than both left- and right-sided diastolic pressures |
| 14 | PAH* | - | - | - | - | - | - | - | Placement of an indwelling pericardial catheter |
| 15 | PAH* | - | Moderate | - | - | - | Serous | - | - |
| 16 | Autoimmune disease | 12 | Large | - | - | - | - | 350 | 350 mL removed |

-: not reported, PC: pericaridocentesis, PAH: pulmonary arterial hypertension

*Pericardial effusion may be attributed to PAH and/or right heart failure.

Supplemental material 11. Table S7

*Major periprocedural complications*

| **ID** | **Major bleeding** | **Iatrogenic complication** | **Hemodynamic instability** | **Vasopressor requirement** | **CPR** | **PDS** | **30-day all-cause mortality** | **Other** |
| --- | --- | --- | --- | --- | --- | --- | --- | --- |
| 1 | 0 | 0 | 0 | 0 | 0 | 0 | 0 | - |
| 2 | 0 | 0 | 0 | 1 | 0 | 0 | 1 | - |
| 3 | 0 | 0 | 1 | 0 | 1 | 0 | 1 | - |
| 4 | 0 | 0 | 1 | 1 | 1 | 1 | 1 | VA - ECMO + Brain Death |
| 5 | 0 | 0 | 0 | 0 | 0 | 0 | 0 | - |
| 6 | 0 | 0 | 0 | 0 | 0 | 0 | 0 | - |
| 7 | 0 | 0 | 0 | 0 | 0 | 0 | 2 | - |
| 8 | 0 | 0 | 0 | 0 | 0 | 0 | 0 | - |
| 9 | 0 | 0 | 1 | 0 | 0 | 0 | 2 | - |
| 10 | 0 | 0 | 1 | 1 | 0 | 0 | 0 | - |
| 11 | 0 | 0 | 0 | 0 | 0 | 0 | 0 | - |
| 12 | 0 | 0 | 0 | 0 | 0 | 0 | 0 | - |
| 13 | 0 | 0 | 0 | 0 | 0 | 0 | 0 | Repeat PC |
| 14 | 0 | 0 | 0 | 0 | 0 | 0 | 0 | - |
| 15 | 0 | 0 | 0 | 0 | 0 | 0 | 0 | - |
| 16 | 0 | 0 | 0 | 0 | 0 | 0 | 0 | - |

-: not reported, CPR: cardiopulmonary resuscitation, PDS: pericardial decompression syndrome, VA-ECMO: veno-arterial extracorporeal membrane oxygenation, PC: pericardiocentesis

Supplemental material 13. Table S8

*Studies including patients with PAH and PE requiring pericardiocentesis as part of a larger cohort or series*

| **Study** | **Cohort** | **PAH + PE + pericardiocentesis patient(s)** | **Complications and mortality** |
| --- | --- | --- | --- |
| 1. Frantz, R.P (2008) | Prospective cohort of 24 patients with PAH undergoing ambulatory hemodynamic monitoring. | 1 | No major or minor complications related to the procedure. |
| 7. Fenstad, E.R (2013) | Retrospective cohort of 577 PAH patients that sought to research PE characteristics, prognosis, and role of drainage. Subgroup analysis of patients who underwent pericardiocentesis. | 14 | Pericardiocenteses were successful without major complications and the 30-day mortality rate was 14% (2 of 14 patients). |
| 9. Hemnes, A.R (2007) | Case series that reported the outcomes of PAH patients with PE and drainage procedures, either pericardiocentesis or surgical pericardial windows. | 4 | Overall mortality rate of 50% (2 of 4 patients) in the pericardiocentesis group. |
| 10. Batal, O (2015) | Retrospective cohort that evaluated the prognostic value of PE on serial transthoracic echocardiograms in 200 PAH patients at a single center. | 2 | One patient presented hypotension and respiratory failure requiring intubation and inotropic support after the procedure. No immediate deaths were attributed to pericardiocentesis. |
| 11. Case, B (2019) | Retrospective cohort studying the safety of pericardiocentesis in 170 patients with pulmonary hypertension. 27 patients had significant pulmonary hypertension and underwent pericardiocentesis, 3 of them had PAH diagnosed with a right heart catheterization. | 3 | No major or minor complications related to the procedure. |
| 14. Ruge, M (2023) | Case series reporting 3 patients who underwent right heart catheterization as an aid in clinical decision-making, the first representing a patient with PAH and PE who underwent pericardiocentesis. The remaining cases are not related to PAH. | 1 | Pericardiocentesis with minimal clinical benefit in a patient who was posteriorly diagnosed with PAH and improved upon starting PAH therapy. No complications associated with the procedure. |
